# Analysis of the Status of Chinese clinical practice guidelines development

**DOI:** 10.1186/1472-6963-12-218

**Published:** 2012-07-25

**Authors:** Zhi-hong Zheng, Shu-qi Cui, Xiao-qin Lu, David Zakus, Wan-nian Liang, Fang Huang, Xiao-na Cao, Ya-li Zhao, Xiao-xia Peng, Ke-qin Rao, Jing Wu

**Affiliations:** 1 Department of Educational Management, Friendship Hospital, Capital Medical University, Beijing 100050, China; 2 Department of Family Medicine, School of Public Health and Family Medicine, Capital Medical University, Beijing 100069, China; 3 Global Health Program, Canadian Public Health Association, Ottawa, Canada; 4 Office of Health Emergency, Ministry of Health, People’s Republic of China, No.1, Xi Zhi Men Wai Nan Road, Beijing 100044, China; 5 Statistical Information Center of Chinese Ministry of Health, Beijing 100044, China; 6 Adjunct Professor, Faculty of Medicine, University of Toronto, Toronto, Canada

**Keywords:** Clinical practice guidelines, Status, AGREE appraisal, Citation, Update

## Abstract

**Background:**

The work of developing clinical practice guidelines began just a little more than ten years ago in China. Up to now, there have been few studies about them.

**Objectives:**

To review and analyze the status of Chinese clinical practice guidelines in 1997–2007.

**Methods:**

All Chinese guidelines from 1997–2007 were collected, and made a regression analysis, and a citation analysis for evaluating the impact of guidelines. To analyze the developing quality, the most influential guidelines were evaluated with AGREE instrument, and each guideline was evaluated to check for any updating. In order to analyze the objective and target population, all guidelines were classified and counted separately according to disease/symptom center, and whether towards specialists or general practitioners.

**Results:**

143 guidelines were collected. An exponential function equation was established for the trend in the number of guidelines. The immediacy index in every year was very low while the average citation rate was not. Both the percentages of highly cited and never cited were high. For the evaluation with AGREE, only the average score of clarity and presentation was high (89.9%); the remaining were much lower. Editorial independence scored 0. Only 27 (18.9%) of 143 guidelines, were found to be evidence-based. Only a few had ever been updated, with an average updating interval of 5.2 years. Only 2.1% were symptom-centered, and only 4.2% were aimed at general practitioners.

**Conclusion:**

Much progress has been obtained for Chinese guidelines development. However, there were still defects, and greater efforts should be made in the future.

## Background

Clinical practice guidelines are systematically developed statements to assist practitioner and patient decisions about appropriate health care for specific clinical circumstances (Institute of Medicine, 1990) [1]. They are expected to promote more consistent, effective and efficient medical practice and to improve health outcomes [2]. A large number of good guidelines have been produced by numerous organizations all over the world, especially in UK, USA, Canada, Australia and New Zealand. The work of developing guidelines began just a little more than ten years ago in China, encouraging progress has been made. But up to now, there have been few studies about them, and know very little about their status. What is their quality like? Are they as scientific and rigorous as the international ones? And how can we improve them. This study aims to describe the status of Chinese guidelines and to identify both successes and defects. We hope to help promote Chinese guidelines development, and to promote Chinese medical practice in general.

## Methods

### Inclusion and exclusion criteria

 (1) Only those guidelines that were developed by authoritative academic organizations were included. Those by individual suggestions of some specialists were excluded.

 (2) Guidelines that were translated from foreign ones were excluded for they were not developed in China.

 (3) Guidelines aimed how to use some medical equipment or how to handle some laboratory test were excluded.

 (4) The time scope of the search was from 1997 to 2007.

### Search strategy

Google Scholar, China National Knowledge Infrastructure (CNKI), Wanfang, Vip and the website of Ministry of Health were searched for all the clinical practice guidelines in China (1997–2007). The key words for the searches included Chinese words for terms such as ‘guidelines’, ‘clinical’, ‘clinical practice’, ‘prevention’, ‘diagnosis’ ‘treatment’, and ‘management’. Chinese Medical Citation Index (CMCI) was searched to compile the citation analysis (1997–2007).

### Trend analysis of clinical guidelines

All the guidelines were recorded on an EXCEL form and were classified by the year when they were developed. A function equation of the number of guidelines according to time was established using SPSS software. According to the equation, the numbers of guidelines in 2008, 2009, and 2010 can be forecasted.

### Citation analysis

CMCI, from 1997 to 2007, was searched to make the citation analysis. But not all the guidelines were collected in it. And the search was done in 2008. Guidelines developed after 2007 have less time to be cited. So the number of the citation would be expected to be less. This is a limitation of the study.

Some citing indices such as the immediacy index, the highly cited rate, never been cited rate, and the average citation rate were calculated separately. The formulae and explanations of them are listed in Table 1.

**Table 1:** The formulae and explanations for calculation of the citing indices

**index**	**formula**	**explanation for character**
immediacy index	= C1/A	‘C1’ means the number of times that all the guidelines were cited in the year when published for the first time.
		‘A’ means the total number of guidelines in the year when published for the first time (the year is the same as one of C1).
high cited rate	= G1/N	‘G1’ means the number of guidelines that were highly cited
		‘G2’ means the number of guidelines that were never cited
never been cited rate	= G2/N	‘C2’ means the number of cited times of all the guidelines being collected for citation analysis.
average citation rate	= C2/N	‘N’ means the number of all the guidelines being selected for citation analysis

For the formula of the immediacy index, the numerator and denominator of the fraction are of the same period, in this study, the year in the formula is designated a calendar year. If the same guidelines were published in different journals, we regard them as one guideline. We only calculated the number of times cited in the year when the guidelines were published for the first time.

Those guidelines which were cited more than 30 times were regarded as highly cited guidelines.

### Appraisal of the guidelines using the AGREE instrument

The highest cited guidelines (having been cited more than 500) were further evaluated using the AGREE I instrument (version 2003) [3] as they had more impact on practice. Two reviewers performed the evaluation. Both reviewers were medical graduates and familiar with the AGREE instrument. Neither of them was provided with financial reimbursement for their work, and none reported any conflict of interest.

Each of these guidelines was evaluated across six domains including: scope and purpose, stakeholder involvement, rigor of development, clarity and presentation, application, and editorial independence. And the standardization of scores (percentage) of them were calculated. Lastly, the average standardization of scores for 7 guidelines for every domain was calculated.

### Analysis of the number of evidence-based guidelines

All the guidelines were appraised whether they met the criterion that it is mentioned in the document about rating the quality of evidence or grading recommendation strength or having referred to allied evidence-based guidelines abroad in the process of guideline’s development. The percentage of them was calculated.

### Analysis of the updating of Chinese clinical guidelines

All the guidelines that had been updated were collected and their percentage was calculated. And the average updating interval was obtained through using the sum of all updated interval divided by the total number of all updated guidelines.

### Analysis of objective and target population

All the guidelines were divided into disease guidelines (disease center) and symptom guidelines (symptom center), and percentages of them were calculated, and those special for general practitioners were calculated.

## Results

### Collection of the guidelines

The total number of documents which were related to guidelines was more than 400. 143 guidelines were selected according to the selection criteria.

### The increasing trend of guidelines amount in China

Some statistics for the number of Chinese guidelines in 1997–2007 were calculated, that are listed in Table 2. An exponential function equation was obtained to describe the trend of the guidelines’ number developed (according to the date affixed to the guidelines document) during last decade.

$$\text{That is}:F\left(t\right)=1.450\times e^{0.290t}.$$

**Table 2:** Amount and citing analysis of Chinese clinical guidelines developed in 1997- 2007

***Year***	**Actual number of guidelines**	**Calculated data of guidelines’ numbers**	**95% confidence interval of calculated data of guidelines’ number**	**Immediacy index**
1997	2	1.938	0.626-5.990	3
1998	3	2.590	0.867-7.726	---*
1999	3	3.461	1.192-10.034	1
2000	3	4.625	1.627-13.134	5
2001	5	6.182	2.202-17.330	5.4
2002	8	8.261	2.956-23.062	0.3
2003	29	11.040	3.934-30.953	2
2004	18	14.755	5.191-41.895	1.04
2005	13	19.719	6.794-57.169	0.71
2006	36	26.353	8.824-78.612	0.7
2007	23	35.218	11.380-108.867	2.75

Note:

Immediacy index is used to measure the adoption speed of guidelines. It equals C1/A as listed in Table 1.

* No guidelines were published in 1998. (All the guidelines developed in 1998 had not been published until 1999) So the immediacy index cannot be calculated in 1998.

‘F(t)’ represents the number of guidelines; ‘t’ is the number of year (as 1 represents year of 1997, and 2 represents1998); ‘e’ is the base number of natural logarithm. F value of the function equation is 48.919 (P = 0.000). R^2^ (Coefficient of determination) =0.845, Adjusted R^2^ = 0.827, indicating a good fitting equation.

All the actual numbers of guidelines are within the confidence interval as shown in Table 2, what further prove the equation is a good fit.

From the function equation, the forecasted number of guidelines in 2008, 2009, and 2010 would be 47, 63, and 84 respectively.

**Figure 1:**
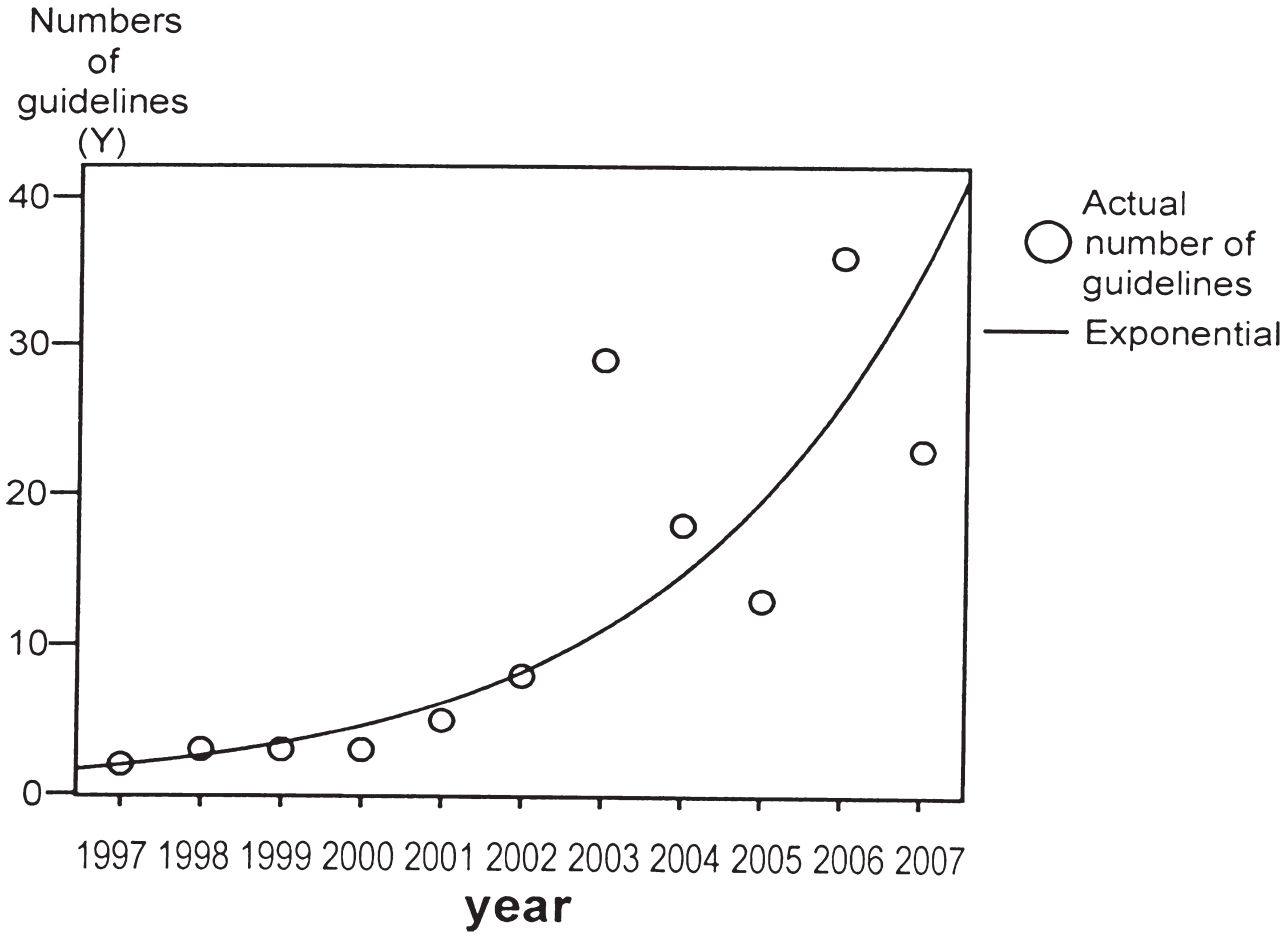
The exponential curve of Chinese clinical guidelines published between 1997 and 2007.

The Figure 1 describes the increasing trend of Chinese clinical guidelines in 1997–2007.

### The citation analysis of clinical guidelines in China

A total of 108 guidelines were collected for the citation analysis. The results are listed as below.

 (1) Immediacy index in every year is listed in Table 2.

 (2) The number of highly cited guidelines was 33 (30.6%). Among them respiratory and cardiology medicine had the most guideline with 12 (11.1%) and 6 (5.6%), respectively.

The three most cited guidelines were the Guidelines for Prevention and Treatment of Bronchial Asthma (produced in 1997) [4], the Guidelines for Diagnosis and Treatment of Chronic Obstructive Pulmonary Disease (COPD) [5], and the Guidelines for Prevention and Treatment of Hypertension in China (produced in 1999) [6]. Their citation frequencies were 1720, 1446, and 685 respectively.

 (3) 20 guidelines were never cited (18.5%).

 (4) The average citation rate was 94.3. Different disciplines had different citation rates. The highest were respiratory medicine (429.9) and cardiology medicine (246.1). The average citation rates of most disciplines were between 2.5 and 86. The rates of some disciplines were very low, even 0 or 1.

### Appraisal of selected guidelines using the AGREE instrument

The standardization of scores (percentage) of six domains are listed in Table 3, as well as average standardization of scores for all 7 guidelines, spearman correlation coefficients between two reviewers and their statistic significance.

**Table 3:** The scores of evaluating 7 guidelines by AGREE instrument

**AGREE Domain**	**Asthma (1997)**^**a**^	**COPD**^**b**^	**Hyper-tension**^**c**^	**AMI**^**d**^	**Asthma (2003)**^**e**^	**HAP**^**f**^	**OSAHS**^**g**^	**Average Score**
scope and purpose (%)	16.7	27.8	66.7	55.6	16.7	72.2	33.3	41.3
stakeholder involvement (%)	4.2	0.0	54.2	12.5	0.0	0.0	0.0	10.1
rigor of development (%)	14.3	16.7	28.6	45.2	14.3	2.4	14.3	19.4
clarity and presentation (%)	95.8	95.8	100.0	83.3	83.3	75.0	95.8	89.9
Applicability (%)	16.7	27.8	50.0	16.7	22.2	11.1	16.7	23.0
editorial independence (%)	0.0	0.0	0.0	0.0	0.0	0.0	0.0	0.0%
Spearman correlation coefficient	0.754	0.600	0.493	0.472	0.569	0.789	0.835	
Sig.(2-tailed)	<0.01	<0.01	<0.05	<0.05	<0.01	<0.01	<0.01	

Note:

a. the Guidelines for Prevention and Treatment of Bronchial Asthma (produced in 1997).

b. the Guidelines for Diagnosis and Treatment of COPD.

c. the Guidelines for Prevention and Treatment of Hypertension in China (produced in 1999).

d. the Guidelines for Diagnosis and Treatment of Acute Myocardial Infarction (AMI) [7].

e. the Guidelines for Prevention and Treatment of Bronchial Asthma (produced in 2003) [8].

f. the Guidelines for Diagnosis and Treatment of Hospital Acquired Pneumonia (HAP) [9].

g. the Guidelines for Diagnosis and Treatment of Obstructive Sleep Apnea/Hypopnea Syndrome (OSAHS-Draft) [10].

For all the domains of the seven guidelines being appraised, only clarity and presentation scored highly (89.9%). The remaining scored much lower including scope and purpose (41.3%), stakeholder involvement (10.1%), rigor of development (19.4%), application (23.0%), and editorial independence (0.0%).

Editorial independence scored 0 because none of the guidelines provided any information about this criterion, as well as not for stakeholder involvement and applicability in most guidelines. For the domain of stakeholder involvement, only one guideline (i.e. Guidelines for Prevention and Treatment of Hypertension in China) scored 54.2% while two others scored 12.5% and 4.2%, the rest all 0. The average score of scope and purpose was better than other domains (except for clarity and presentation). Of this domain, the item of considering benefits, side effects and risks scored well in most guidelines. However, there was not any information provided in any guidelines for the items of external review and updating. The average score of rigor of development was not satisfactory either (<30%). For the criterion of selecting the evidence only one guideline scored 4, while all the others scored 1. Developers of most guidelines had not rigorously evaluated evidence by themselves.

### The amount of evidence-based guidelines

Only 27 (18.9%) of 143 guidelines, were found to be evidence-based. Some of them referred only to the evidenced-based ones from abroad. The developers had not rigorously assessed the evidence by themselves. And there were many guidelines in which the recommendations were not derived from formal consensus methods such as Delphi or Nominal Group Technique.

### Updating of Chinese clinical guidelines

Only 11 guidelines had been updated. For those the updating interval was between 2 and 10 years, with average interval of 5.2 years.

### Objective and target population of guidelines in China

Of all 143 guidelines, 140 (97.9%) were aimed at diseases, while only three (2.1%) aimed at symptoms, and only six (4.2%) special for general practitioners. There were not any guidelines aimed at referral between general practitioners and specialists.

## Discussion

### Search limitation

The study began 4 years ago, so the search for guidelines is just from 1997 to 2007. After that, much time has been used to write and edit this article. And the data has not been updated, which can not reflect the status of guidelines in China of the last 4 years. This is a limitation of the study.

### Progress of the amount of Chinese guidelines

There has been an exponential increase in the number of Chinese guidelines during the last decade. Much progress had been made. Because the Chinese Society of Rheumatic Disease developed many guidelines for rheumatic disease in 2003 (t = 7), and the Chinese Society of Osteoporosis, Bone and Mineral Disease also developed many in 2006 (t = 10), a large increase in the number of guidelines was obtained in those two years.

### The impact on medical practice of Chinese guidelines was acceptable but not the same in all disciplines. The adoption speed of guidelines was not rapid

The Average Citation Rate is the number of times that all the guidelines having been cited divided by the number of all guidelines. This can reflect a journal’s influence. It is generally said that a journal has high academic impact when the average citation rate is high. The Immediacy Index is the number of times that all the guidelines were cited divided by the total number of guidelines published in the year when the guidelines were published for the first time. The index introduced by Garfield is used to measure the adoption speed. Good journals and good papers will be read and adopted quickly by many persons [11]. These two indexes were adopted in this study to reflect Chinese guidelines’ impact to some extent (not equate), as well as dissemination and utilization.

The results showed that the average citation rate of Chinese clinical guidelines was rather high (>30). That means the guidelines impact was acceptable. But the Immediacy Index of every year was low what indicated their adoption speed was not rapid. More attention should therefore be given to the dissemination. We can also see the impact difference of the guidelines as well as their quality from the uneven of these cited numbers.

It must be admitted that the citation indexes could not completely reflect the guidelines influence as being cited does not equal importance. And as most Chinese guidelines did not include references, the number of citations of other papers used for their development has not been calculated. These are all the limitations of this study.

### Appraisals to guidelines development with the AGREE instrument; merits and shortcomings

There are accepted guideline evaluation instruments developed by different countries. Examples include the IOM’s “Provisional Instrument for Assessing Clinical Practice Guidelines” (IOM instrument), the “Method for Evaluating Research and Guidelines Evidence” (MERGE instrument), Cluzeau et al’s “Appraisal Instrument for Clinical Guidelines” (Cluzeau instrument), and Shaneyfelt et al’s methodological appraisal instrument (Shaneyfelt instrument). Of all these instruments, a study showed that The Cluzeau instrument was the most well developed and had been tested and described as a reliable and valid method of guideline evaluation [12].

Based on the Cluzeau instrument, AGREE instrument was developed [12]. Up to now, it is the only guidelines instrument to have undergone extensive international validity assessment [13]. But there is a limitation that it does not evaluate the quality of evidence, what is better covered by GRADE, an approach to develop and present recommendations for management of patients through rating quality of evidence and grading strength of recommendation [14-16].

Many other studies using the AGREE instrument for guideline evaluation reported the lowest scores in the applicability domain, and the highest in the scope and purpose domain [17]. Contrasting to that, the result of this study show that the lowest score is in the Editorial independence, and the highest is in the clarity and presentation in China. This result helps recognizing the defects in the development process of Chinese guidelines. It should pay more attention to the domains of editorial independence in the future, so as to stakeholder involvement, and applicability. As for items that the external review, updating, evidence selecting criteria and evidence evaluation should also receive more attention.

### The method of developing guidelines in China is less scientific and lags behind the international level

Evidence-based guidelines apply the principles of evidence-based medicine to the process of guideline development. The first step is defining the clinical question. This is followed by defining the eligibility criteria for the studies. A systematic search of the literature is then conducted and the evidence is evaluated. In developing recommendations, the likely benefits, risks, inconvenience and costs associated with each treatment must be considered in addition to addressing patients’ underlying values and preferences. The quality of the data supporting the recommendations is evaluated and is reflected in a grading system that describes the strength of the recommendation and the quality of the supporting evidence. This process ultimately results in the systematic development of recommendations that incorporate evidence with patients’ preferences and values and indicates the quality of the evidence [18].

However, in China, there have not been any criteria on how guidelines should be developed. The method of developing Chinese guidelines is less scientific and lags behind the international level. From the results it can be seen that most Chinese guidelines not with the evidence-based method. This makes it difficult to ensure quality. In addition, most Chinese ones have not listed references of important evidence. The user is not therefore able to examine the validity of the recommendations.

### The interval and timeliness of Chinese guidelines’ updating

A ‘valid guideline should be up-to-date. Possible consequences of using out-of-date guidelines include a clinician’s use of diagnostic studies or treatments that do not provide the best-known outcomes [19]. So a good guideline should have specific updating timetable. R.E. Burton calculated that the half-life of a document in biology and medicine is three years based on Burton-Kebler’s aging equation for science and technology documents. Another study also suggested that as a general rule, guidelines should be reassessed for validity every three years [20].

Of all Chinese guidelines developed between 1997–2007, only a few have been updated. And of them the average updating interval is more than three years, falling short of international standards. Because the search was done in 2008, some guidelines developed after 2004 have less time to be updated. So the number of updated guidelines would be expected to be less. This is also a limitation of the study.

### Scope of Chinese guideline’s objective and target population are not wide enough

There is shortage of Chinese guidelines aimed at general practitioners and referral to specialist care. There is also a shortage of aiming at symptoms. This is behind international levels. For instance, in the case of ‘cough’, there is only one guidelines named “Guidelines for Cough Diagnosis and Treatment (Draft) in China”, while there are 24 in America. While general practice is developing in China, more and more general practitioners require guidelines for them, especially of referral and symptom center. Those guidelines should be assigned a priority.

## Conclusion

This study shows that from amount to quality, much progress has been made of guidelines in China. But it was uneven in different disciplines. There were some problems in the development process and dissemination which should be solved for better effect on practice. The guidelines involving various objectives and target population should be developed in the future to help clinical practice.

## Competing interests

The authors declare that they have no competing interests.

## Authors’ contributions

Z-h Z have made substantial contributions to design, acquisition and analysis of data and writing the manuscript, S-q C have made substantial contributions to conception and design, X-q L have made the substantial contributions to design and modify the manuscript, DZ revised the manuscript, W-n L have participated in the drafting the manuscript, FH have contribution to analysis and interpretation of data, X-n C have contribution to acquisition of data, Y-l Z have participated in the design, X-x P have contribution to analysis and interpretation of data, K-q R have participated in the drafting the manuscript, J W have contribution to analysis and interpretation of data. All authors read and approved the final manuscript.

## Pre-publication history

The pre-publication history for this paper can be accessed here:

http://www.biomedcentral.com/1472-6963/12/218/prepub
